# A spatial database of health facilities managed by the public health sector in sub Saharan Africa

**DOI:** 10.1038/s41597-019-0142-2

**Published:** 2019-07-25

**Authors:** Joseph Maina, Paul O. Ouma, Peter M. Macharia, Victor A. Alegana, Benard Mitto, Ibrahima Socé Fall, Abdisalan M. Noor, Robert W. Snow, Emelda A. Okiro

**Affiliations:** 1 Population Health Unit, Kenya Medical Research Institute - Wellcome Trust Research Programme, P.O. Box 43640-00100, Nairobi, Kenya; 2 Geography and Environmental Science, University of Southampton, Southampton, UK; 3 World Health Organization, Regional Office for Africa, Brazzaville, Congo; 4 Global Malaria Programme, World Health Organization, Geneva, Switzerland; 5 Centre for Tropical Medicine and Global Health, Nuffield Department of Clinical Medicine, University of Oxford, Oxford, UK; 6 Faculty of Science and Technology, Lancaster University, LA1 4YR, UK

**Keywords:** Health services, Developing world, Geography

## Abstract

Health facilities form a central component of health systems, providing curative and preventative services and structured to allow referral through a pyramid of increasingly complex service provision. Access to health care is a complex and multidimensional concept, however, in its most narrow sense, it refers to geographic availability. Linking health facilities to populations has been a traditional per capita index of heath care coverage, however, with locations of health facilities and higher resolution population data, Geographic Information Systems allow for a more refined metric of health access, define geographic inequalities in service provision and inform planning. Maximizing the value of spatial heath access requires a complete census of providers and their locations. To-date there has not been a single, geo-referenced and comprehensive public health facility database for sub-Saharan Africa. We have assembled national master health facility lists from a variety of government and non-government sources from 50 countries and islands in sub Saharan Africa and used multiple geocoding methods to provide a comprehensive spatial inventory of 98,745 public health facilities.

## Abstract (summary)

**Table:** 

Design Type(s)	data integration objective • database creation objective
Measurement Type(s)	Healthcare Facility
Technology Type(s)	digital curation
Factor Type(s)	geographic location • Facility • organization
Sample Characteristic(s)	Sub-Saharan Africa • public infrastructure

Machine-accessible metadata file describing the reported data (ISA-Tab format)

## Background and Summary

Defining the location of health services in relation to the communities they are intended to serve is the cornerstone of health system planning, ensuring the right services are accessible to the population and that no one is geographically marginalized from essential services. Across sub-Saharan Africa (SSA), health services are offered with increasing levels of medical sophistication, from community providers who handle basic care to hospitals which play a critical role in providing emergency care^1–3^. However, these services are not accessible to everyone equally^4^. This inability to reach quality assured health services able to provide life-saving interventions contributes to the sustained high burden of communicable and non-communicable disease morbidity and mortality in SSA relative to other regions of the world^5,6^. The Global health agenda now aims to ensure Universal Health Coverage (UHC), to ensure that all people obtain the health services they need and underpins the health-related Sustainable Development Goals. Defining and planning for universal equity in access to health services demands better data on the location of both services and populations they are intended to serve.

While countries have been encouraged to develop health Master Facility Lists (MFLs)^7,8^ and censuses of service provision^9^, this has not been universal and most inventories lack coordinates. In Kenya in 2003, we began an exercise to compile and geocode a single, public health MFL from a variety of government, non-governmental (NGO), community-based (CBO) and faith-based organization (FBO) listings^10^, the first time a national map of service providers had been developed since 1959^11^. Over the next 15 years we extended this approach across other countries in SSA, coinciding with a regional impetus to improve master health facility lists^7,8^.

Access to health care is a complex and multidimensional concept and can be defined in a variety of ways, however, in its most basic sense, it refers to geographic availability. Improved data metrics on health access demands at the very least a knowledge of where service providers are in relation to populations. Provider-to-population ratios were used to estimate availability in 1970s and earlier^12–14^, then the emergence of Geographic information systems (GIS) in 1980s and 1990s led to the evolution of distance as a metric^15–17^ while network analyses, floating catchment area methods and cost-distance analyses are more recent^18–21^. By identifying top tier hospitals and lower tier facilities, it is now possible to estimate specific access to emergency care and surgeries^18,22–24^. Other extensions are application in defining access to Basic and Comprehensive Emergency Obstetric and Neonatal Care (BEmONC/EmONC) services^25^, evaluating public health sector service delivery^26^ and community-based interventions^27–29^. In the future, it is likely that this will be extended to understanding health care optimization. Georeferenced health service inventories, beyond defining equitable access, increase the epidemiological value of defining the evolution of emerging, epidemic pathogens^30^, antimicrobial resistance, and defining sub-national disease epidemiology as well as improving the potential of routine health data use^31^.

Notwithstanding previous efforts to extract health service providers from OpenStreetMap complimented by volunteered information (https://www.healthsites.io), there is no single, georeferenced and comprehensive public health facility inventory for sub-Saharan Africa. Building on a previous audit of public hospitals in sub Saharan Africa^18^, we provide a geo-coded inventory of 50 countries in sub-Saharan Africa, provided as an open-access resource. We have focused on public health facilities managed by government, local authorities, FBOs and NGOs. A wide variety of primary sources of information were consulted, from websites and data portals of ministries of health, national and international organizations, health sector reports, to sourcing data through personal communications. Facility types and numbers for each country were validated using reported data in national health sector strategic plans and other health sector reports.

## Methods

### Data sources

**Fig. 1:**
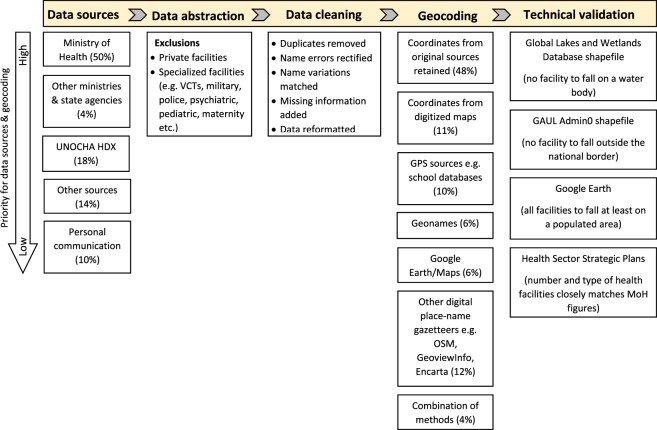
The methodological framework applied in assembling each country’s Master Facility List. Percentages for “Data sources” refer to proportion of the 50 countries with that source as the main or only source of data, while percentages under “Geocoding” refer to the proportion of all 98,745 health facilities.

We undertook a data assembly process to compile a comprehensive health facility database across SSA, starting with Kenya in 2004^10,32^, and expanding to the rest of SSA between 2012 and 2018. Country-specific public health facility databases were developed through a systematic and iterative process of data assembly from a variety of sources, data abstraction, geocoding and technical validation of the data. For each country, the Ministry of Health (MoH) website was the first source of authoritative MFLs consulted. This was done through online searches and downloads of MFLs or maps hosted on the MoH website or on data portals managed by the MoH such as the National Health Map (*Carte Sanitaire*), health facility registries and online Health Management Information Systems (HMIS) including District Health Information Systems version 2 (DHIS2). In some instances, MoH publications with information on facility lists or maps were used. Occasionally, while the MoH sources did not have comprehensive lists of facilities or maps, other government agencies’ websites, for example national statistical agencies, hosted health facility listings and these were consulted. Where the governmental ministries and agencies had no facility listing information or was inadequate, other sources of data were consulted, including data hosted by the United Nations Office for the Coordination of Humanitarian Affairs’ (UNOCHA) Humanitarian Data Exchange (HDX) portal and websites of other UN agencies, and where relevant, websites of FBOs and NGOs working in each country. For countries where no health facility data were available online, we made attempts to directly contact people working with National Malaria Control Programmes, World Health Organization or other health-oriented agencies to aid in locating health facility listings. Often, more than one source of data was consulted for each country and assembled into a single list as illustrated in Online-only Table 1 and Fig. 1.

Locations of existing health facility data for sub-Saharan African countries are fragmented. Half of the 50 countries had an MoH website/HMIS/data portal as the main source of data used; 4 (8%) had other governmental bodies, mainly national statistical agencies, as the main data source; 9 (18%) countries had UNOCHA HDX as the main data source, while we used other sources like NGO and FBO websites for 7 (14%) countries. The main source of data for the remaining 5 countries (10%) is unpublished data obtained through personal communication. In total, 93 different sources of data were consulted to develop the first sub-Saharan Africa geocoded inventory of health service providers across all levels of service provision (Online-only Table 1).

We have classified the 50 countries into 4 broad categories according to each country’s main source of health facility data consulted (Online-only Table 1). We have treated Zanzibar as a separate entity from mainland Tanzania as it has a separate health system. The first category includes 17 countries (Burundi, Central African Republic, Ghana, Guinea, Liberia, Malawi, Mali, Mozambique, Namibia, Nigeria, Rwanda, Sierra Leone, Somalia, South Africa, South Sudan, Tanzania and Zimbabwe) that have an online list of health facilities and for which data are available either fully or near fully geocoded. The second category includes 11 countries (Botswana, Chad, Comoros, Gambia, Kenya, Lesotho, Senegal, Seychelles, Togo, Uganda and Zambia) that had MFLs available online but lacked coordinates and where a process of geocoding was undertaken. The third category includes 17 countries (Benin, Burkina Faso, Cameroon, Cape Verde, Cote d’Ivoire, Djibouti, Equatorial Guinea, Eritrea, eSwatini, Ethiopia, Gabon, Guinea-Bissau, Mauritania, Mauritius, Niger, São Tomé & Príncipe and Zanzibar) that had maps available online where a facility list was created through a process of onscreen digitization of locations in a GIS platform. The fourth category are 5 countries (Angola, Congo, Democratic Republic of Congo, Madagascar and Sudan) where adequate data were not available online, and for which we relied on personal communication and in country contacts to acquire these data. We could not locate data for lower tier facilities for Guinea-Bissau and Sudan, and for 10 provinces in Angola (Online-only Table 1).

### Data abstraction

Broadly, there are two main categories of health care delivery systems across sub-Saharan Africa - public and private. Private includes private-not-for-profit and private-for-profit^33^. We have focused on the public and private-not-for-profit sectors where health facilities are managed by the MoH, local authorities, NGOs, FBOs and CBOs. Private health facilities, though important in extending provision of health services, were excluded as previous audits of MFLs reveal that private sector facilities are often under-represented in MoH registries, located in urban centres, accessible only to those able to afford services, unregulated and do not often feature in MoH commodity distribution systems^17^. Their exclusion was a pragmatic decision based on difficulties with enumeration within the private sector^10,32^ and the complexity of its structural and organizational system^34–37^. Enumerating and regulating this sector has remained a challenge in most countries in sub-Saharan Africa. The private sector was difficult to audit despite extensive searches from multiple sources, where available, completeness varied wildly from country to country, and most of the facilities were often based in large cities and smaller urban areas. Our focus, therefore, has been on providers of public health sector services that cover services including expanded immunization programmes, health data surveillance and receive government funding to provide services to the general population. We have made a single exception to this rule, in Botswana, where many private company-owned health service providers also provide care to the general public and are more formally integrated into the government health system^38^. Across all countries, we excluded government facilities serving special groups, for example prisons, armed services, police, schools, universities and government service clinics. These services are often not available to the general population. Further exclusions were made to remove specialized care service providers such as blood transfusion centres, HIV Voluntary Counselling and Testing (VCT) centres, maternity and nursing homes, family planning clinics and specialist facilities (e.g. dental, eye, psychiatric, tuberculosis, rehabilitation, ophthalmic) (Fig. 1). The spatial location of these services is equally important for health system planning but were not universally documented across national health facility listings. Our ambition was to provide a geo-coded inventory of operational facilities providing broad clinical care services to the general population. It was notable across several data sources that not all facilities were deemed operational, and those labelled as “under construction” or “closed” were excluded. In total, 98,745 public health facilities were assembled for 50 countries in sub Saharan Africa.

### Data cleaning

From all sources a single set of minimal descriptive data was possible for each facility: first level administrative unit, facility name, facility type, and details on facility ownership. A common feature across all databases was the presence of duplicates and these were identified by a careful review of each country’s list and where identified were discarded. For facilities without names, we adopted names of the smallest administrative units i.e. wards, towns or villages, in which they are located if these were included in the original lists, with the assumption being that it is likely that a public facility has same naming orientation as the ward, town or village its located in. For those without names and information on admin units but with coordinates, we adopted the name of the nearest populated place based on calculated relative distances. In instances where information on facility ownership was missing an attempt was made to assign ownership based on the facility name e.g. all facilities that had a Christian name or church name such as Saint, protestant, mission, evangelical or Islamic were assigned FBO ownership, facility names with NGO names were classified as NGO-owned while the rest were assigned government ownership.

The size and definition of level of health facilities varied considerably within and between countries (Online-only Table 1). There doesn’t exists a universal standardized definition of facility types making cross country comparisons difficult, and definitions provided in national health policies varied between countries hence while we are relatively confident in the hospital definition used here, the definition for subsequent tiers were harder to reconcile between countries. We have therefore elected to include the information as shown in primary data sources consulted (Online-only Table 2).

### Geocoding

Facilities that had GPS coordinates from original data sources were 47,805 (48%), while 10,759 (11%) coordinates were obtained from digitizing health facility maps. For the remaining 40,181 (41%) health facilities with missing coordinates, we used a variety of existing location data and digital resources for geocoding. As a priority, where Global Positioning Systems (GPS) data for previously mapped infrastructure like schools were available, we used these to match to names of the health facilities in the same administrative zones to assign coordinates (n = 9,656; 10%). The most useful digital resources were Geonames digital place name gazetteer (n = 6,270; 6%), Google Earth/Maps (n = 5,672; 6%) and other digital place name gazetteers including Encarta, OpenStreetMap, HereMaps, Bingmaps, iTouchMap and Fallingrain (n = 11,925; 12%) (Fig. 1). A comprehensive list of all digital place name gazetteers used is presented in Table 1. Matching facility names to place names proved difficult in some situations. Although digital gazetteers strive to realize standard nomenclatures and unique naming strategies, these are difficult to achieve at national levels where spellings change between authors, overtime and where the same names are replicated across different places in the country^39^. Since all data were stored in a standard format in MS Excel (Microsoft, Redmond, USA), where maps were available, facility locations were digitized in ArcGIS 10.5 (ESRI, Redlands, USA), attribute information captured and converted to MS Excel.

**Table 1:** List of all digital place-name gazetteers and software used to geo-position health facilities.

Name	Link/reference
Geonames	http://www.geonames.org/
Google Earth	Google, Mountain View, California, USA
Google Maps	https://www.google.com/maps
Microsoft Encarta	Microsoft, Redmond, Washington, USA
OpenStreetMap	https://www.openstreetmap.org/
Fallingrain Global Gazetteer	http://www.fallingrain.com/world/index.html
NGA country gazetteers	http://geonames.nga.mil/gns/html/namefiles.html
Geoview country portal	http://geoview.info/
IslamicFinder	http://www.islamicfinder.org/prayerDetail.php
UNOCHA HDX	https://data.humdata.org/dataset?sort=metadata_modified+desc
Alexandria Digital Library	http://www.alexandria.ucsb.edu
ILRI Geoportal	http://data.ilri.org/geoportal/catalog/main/home.page
ArcGIS online	http://www.arcgis.com/home/webmap/viewer.html?useExisting=1
African Data Sampler	http://gcmd.nasa.gov/records/GCMD_ADS_WRI.html
MapCarta	http://mapcarta.com/
Maplandia	http://www.maplandia.com/
Global geodatabase-cities	http://www.geodatasource.com/
VMAP0	http://earth-info.nga.mil/publications/vmap0.html
Bing Maps	https://www.bing.com/maps/
Here Maps	https://wego.here.com/
Old maps online	http://www.oldmapsonline.org/
Mapcruzin	http://www.mapcruzin.com/free-africa-arcgis-maps-shapefiles.htm
CIESIN	http://sedac.ciesin.columbia.edu/data/set/grump-v1-settlement-points
FreeGIS data	https://freegisdata.rtwilson.com/
ILRI Geoportal	http://data.ilri.org/geoportal/catalog/main/home.page
GeoDataSource: World Major Cities Database	https://www.geodatasource.com/
GetaMap	http://fr.getamap.org/AF-afrique.html
Geoba.se	http://www.geoba.se/index.php
iTouchMap	https://itouchmap.com/latlong.html
Citipedia.info	https://www.citipedia.info/continent/general/Africa
GeoPostCodes	http://www.geopostcodes.com/data
Geody	https://www.geody.com/?world=terra

**Fig. 2:**
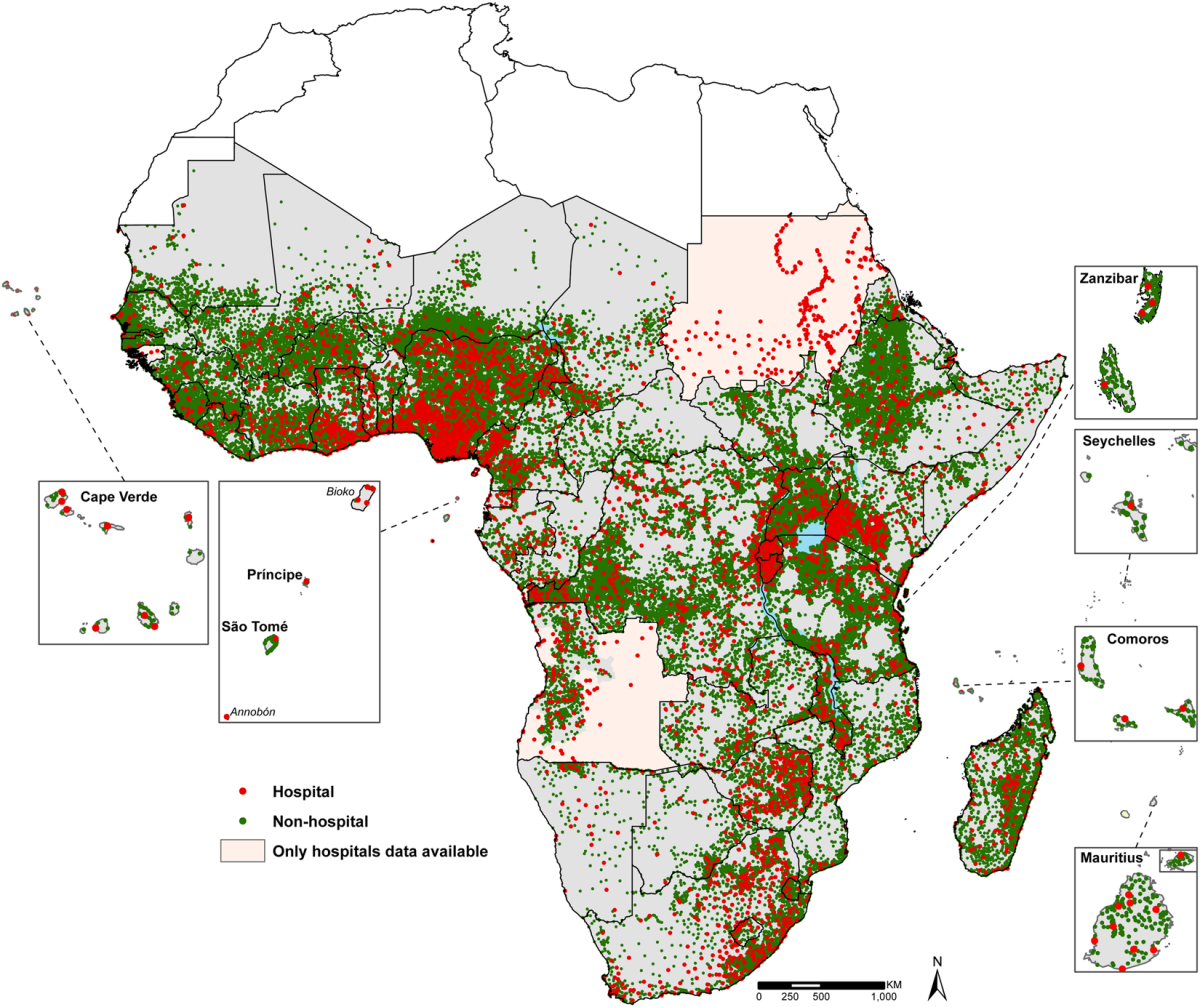
The distribution of 96,395 geocoded public health facilities in sub Saharan Africa. Red dots represent hospitals (n = 4,930) and green dots represent non-hospitals (n = 91,465). Facilities have been mapped on Global Administrative Unit Layers (GAUL) 2008 admin0 boundaries (http://www.fao.org/geonetwork/srv/en/main.home).

For facilities that could not be geo-located using any of the above resources, we used a combination of sources and methods to geocode these (n = 4,308; 4%). This involved finding descriptions of locations of villages matching health facility names in journal papers and news articles and using this information to triangulate these locations in Google Earth, or using shapefiles of the smallest administrative divisions of a country, matching these with the same administrative units in the facility databases and placing the facility location on a populated place in Google Earth, but only in administrative units that were roughly 5 square kilometers in area to minimize the location error. Within small administrative units in Google Earth, populated places were not always obvious to locate visually so we located them by tracing footpaths, roads, rivers or streams that led up to populated places. Facilities that could not be geocoded using any of the sources in Table 1 in the final SSA MFL are 2,350 (2%). Figure 2 shows the location of the geocoded 96,395 public health facilities in sub Saharan Africa.

## Data Records

The geocoded master facility list described here has been made publicly and freely available through both the figshare repository^40^ and through the World Health Organization’s Global Malaria Programme (https://www.who.int/malaria/areas/surveillance/public-sector-health-facilities-ss-africa/en/) in Microsoft Excel format. While the dataset available through figshare^40^ will be preserved in its published form, the one available through the World Health Organization’s Global Malaria Programme website will be updated through further in-depth validation and updating in collaboration with individual countries. The Database includes data from 50 countries in sub-Saharan Africa. Each data record represents a health facility and has 8 descriptive variables – Location identifiers including: country, first level administrative division, latitude, longitude and LL source (source of the coordinates). Coordinates are rounded off to four decimal places for uniformity, allowing an accuracy of 5–10 metres in decimal degrees coordinate format. We have included only the first level administrative divisions for each country that were in the primary data sources as they are least ambiguous and do not change much over time unlike second level administrative divisions which can change rapidly and are not consistent over time for most countries. The other pertinent details are the facility name, facility type and facility ownership. Facility types definitions vary between countries (Online-only Table 2), for example, a health centre in Kenya may not necessarily equal to a health centre in Sierra Leone in terms of services offered. Ownership expresses who owns or manages a facility e.g. MoH (government), local authority, NGO, FBO or CBO. Health facility coordinates are given in decimal degrees format in the World Geodetic System 1984 (WGS84) coordinate system.

## Technical Validation

### Heath facility locations

Facility locations were validated in ArcGIS using two datasets and by visual inspection in Google Earth. In ArcGIS, the Spatial Join and Select by Location tools were used to identify facilities that fell slightly off international boundaries or slightly outside of their correct region and/or district. The Global Lakes and Wetlands Database (GLWD)^41^ shapefile was used to ensure facilities were within defined land areas. While in theory GPS coordinates should represent an unambiguous spatial location, these required careful re-evaluation to ensure that facility locations were on their true position on the Earth’s surface. We therefore routinely verified all GPS data from all sources using place names and/or Google Earth to ensure coordinates were located on populated places. Despite 47,805 (48%) facilities in the original lists having coordinates, some of the these plotted in wrong districts, on water bodies and or outside country boundaries. These were assigned new coordinates using Google Earth and minor shifts made in ArcGIS.

### Service delivery levels and number of health facilities

Health facilities levels are defined based on the complexity of services they offer. Health facilities types, specifically at the primary level, vary between countries in their definitions, specialization, size of populations they serve and the essential services they provide, infrastructure and staffing. Health centres, medical centres, polyclinics, health posts, dispensaries, clinics, health huts, health units etc. may have similar functions but equally may represent different levels of service provision between countries. To cross-reference the completeness of our national inventories of facilities and to understand this variation, we first identified and defined service provision and health facilities at each level based on information from health sector policies and strategic plans for each country. We compared this against information in our database and for most countries, there was good concordance across all levels of service provision. Secondly, the number of health facilities at each service provision level for each country were compared with the corresponding number of facilities reported in the most current Health Sector Strategic Plans (HSSP) and other health sector reports and these were found to be largely similar with minor variations (Online-only Table 2). Most of these variations are partly because HSSPs and other health sector reports usually under-report mission and NGO facilities or do not at all, and in some instances, there was temporal misalignment between the data included in the database and the HSSPs consulted. While this inventory serves as a useful entry point to future facility censuses in Africa, the accuracy and completeness of this resource now requires further country and regional level validation.

## Usage Notes

The definitions of health facility levels provided in national health policies and across country specific MFLs varied between countries hence the arbitrariness of health facility definitions used here. This has been noted previously^8^ and demands development of uniform health service delivery definitions across Africa. Importantly, indicators of service availability cannot accurately reflect access to and utilization of services. Hence service accessibility must be accompanied with improved quality of care at facilities. Accessibility on its own does not by itself guarantee that services are available^42^. Defining the scope, service provision and laboratory capacities and optimal catchment populations for health care providers should therefore be a priority.

We have elected to exclude the facility codes provided in the original sources because, though important, we are unsure of the fidelity of these facility codes as unique facility identifiers in each country without further investigation, and these codes were absent from most of the original data sources we consulted. While we acknowledge the significance of facility codes in managing database updates and providing linkages to other datasets, we encourage countries to create a uniform national coding system for facilities to be used by all stakeholders. For now, future updates and linkage to this dataset will be possible through name and admin units matching, while facility type and ownership can also be used to refine the matching.

The private sector has grown significantly over the last two decades in many countries in Africa, and they will provide an important role in achieving universal health coverage. However, enumerating and regulating this sector has remained a challenge in most countries in sub-Saharan Africa and are difficult to audit within current master health facility lists. Future iterations of the African database of public service providers released here should aim to include private sector providers following improvements in their national enumerations and auditing.

In addition to the data release through the figshare repository^40^ this dataset will simultaneously be hosted by the World Health Organization’s Global Malaria Programme (WHO-GMP) (http://www.who.int/malaria/areas/surveillance/public-sector-health-facilities-ss-africa/en) who will henceforth facilitate further in-depth validation and updating in collaboration with individual countries covered. This will also bring the possibility of assigning the facility codes for future updating, data linkages and comparisons. We have focused this work on SSA countries as this is the region our previous work has focused on, is most lacking with respect to comprehensive MFLs and it’s the region under the World Health Organizations’ Regional Office for Africa, who we have collaborated with on this work.

We recognize that, despite our best efforts, this dataset is incomplete and will change over time. As such, we encourage readers to alert us via email to additional and/or newly available data so that we may add them, if appropriate.

### ISA-Tab metadata file

Download metadata file

## Data Availability

The data assembly was performed using MS Excel (Microsoft, Redmond, USA). The datasets included in this paper were manipulated using ESRI ArcGIS v10.5 and Google Earth V7.3.2.5776. Where maps were available, facility locations were digitized in ArcGIS, attribute information captured and converted to MS Excel.

## Online-only Tables

**Online only Table 1:** Description of data sources, including personal communication, by country and year data acquired and total number of public health facilities assembled for each country. The data sources are listed chronologically with the main data sources listed first. Notes indicate details of the final state of the health facility list in each country after data cleaning, abstraction and geocoding.

Country	Sources of database	Year	MFL available online & geocoded	MFL available online & not geocoded	Maps available online & digitized	Total number of health facilities assembled (% geocoded)	Notes
Angola	• Personal communication	2013 & 2017	N	N	N	1,575 (91)	Non-hospitals (Lower level facilities) data missing in 10 provinces; 140 missing coordinates
Benin	• (https://docplayer.fr/75764654-Republique-du-benin-ministere-de-la-sante.html) • Personal communication	2017 2014	N	N	Y	819 (100)	
Botswana	• (http://www.gov.bw/en/Ministries–Authorities/Ministries/MinistryofHealth-MOH/About-MOH/About-MOH/)	2015	N	Y	N	624 (96)	22 missing coordinates
Burkina Faso	• (http://www.cns.bf/IMG/pdf/carte_sanitaire_2010.pdf) • Personal communication	2012 2012	N	N	Y	1,721 (100)	
Burundi	• (https://insp.bi/wp-content/uploads/2018/04/Rapport-FOSA-d%C3%A9finitif-final-23-01-2014.pdf)	2016	Y	N	N	665 (100)	
Cameroon	• (https://www.dhis-minsante-cm.org/portal/) • Personal communication	2017 2014	N	N	Y	3,061 (99)	42 missing coordinates
Cape Verde	• (https://www.minsaude.gov.cv/index.php/documentosite/238-plano-nacional-de-desenvolvimento-sanitario-vol-i/file) • (http://www.caboverde-info.com/Sociedade)	2016 2016	N	N	Y	66 (98)	1 missing coordinate
Central African Republic	• (https://www.healthsites.io/) • Personal communication	2018 2014	Y	N	N	555 (98)	10 missing coordinates
Chad	• (https://www.humanitarianresponse.info/sites/www.humanitarianresponse.info/files/documents/files/tcd_rpt_annuairesstatistiquessanitaires_ministeresante_2013.pdf) • (https://data.humdata.org/dataset/chad-gis-geodatabase) • Personal communication	2013 2016 2013	N	Y	N	1,283 (96)	54 missing coordinates
Comoros	• (https://docplayer.fr/29010911-Profil-du-systeme-de-sante.html)	2016	N	Y	N	66 (98)	1 missing coordinate
Congo	• Personal communication	2014	N	N	N	328 (98)	7 missing coordinates
Cote d’Ivoire	• (http://www.synamepci.org/synamep/doc/CARTE_SANITAIRE/CARTE_SANITAIRE_2010_22.02.12.VF.pdf) • Personal communication	2014 2014	N	N	Y	1792 (99)	25 missing coordinates
Democratic Republic of Congo (DRC)	• Personal communication • (https://web-archive.lshtm.ac.uk/www.linkmalaria.org/sites/link/files/content/country/profiles/DRC%20Final%20Report%20June%20(240614).pdf) • (http://www.finddiagnostics.org/programs/hat-ond/hat/health_facilities.html)	2014, 2017, 2018 2014 2013	N	N	N	14,586 (100)	
Djibouti	• (http://www.sante.gouv.dj/Carte%20sanitaires.htm) • (http://apps.who.int/medicinedocs/documents/s17292e/s17292e.pdf)	2012 2016	N	N	Y	66 (98)	1 missing coordinate
Equatorial Guinea	• Personal communication	2017 2017	N	N	Y	47 (100)	
Eritrea	• (https://www.afro.who.int/sites/default/files/2017-05/eritrea-health-mdgs-report-2014.pdf) • Personal communication	2014 2016	N	N	Y	269 (100)	
eSwatini	• (https://www.shbcare.org/docs/SAM.pdf) • Published paper^31^ • Personal communication	2018 2014 2013	N	N	Y	135 (98)	3 missing coordinates
Ethiopia	• Ethiopia Central Statistical Agency^43^ • (https://ideas.lshtm.ac.uk/wp-content/uploads/2017/08/Berhanu_DIPH_Ethiopia_17Sept2013.pdf)	2015 2015	N	N	Y	5,215 (99)	39 missing coordinates
Gabon	• (http://csgabon.info/file/f2/Annuaire%20statistique%20sante%202011.pdf) • Personal communication	2011 2013	N	N	Y	542 (98)	10 missing coordinates
Gambia	• (http://www.moh.gov.gm/public) • (https://www.mindbank.info/item/5970)	2015	N	Y	N	103 (98)	2 missing coordinates
Ghana	(http://data.gov.gh/dataset/health-facilities)	2017	Y	N	N	1,960 (96)	82 missing coordinates
Guinea	(https://data.hdx.rwlabs.org/dataset/health-centers-data-base)	2015	Y	N	N	1,746 (87)	225 missing coordinates
Guinea-Bissau	• Google Earth^44^	2017	N	N	Y	8 (100)	Only hospitals data available
Kenya	• (http://kmhfl.health.go.ke/) • (https://hiskenya.org/dhis-web-commons/security/login.action) • Published papers^10,32^	2016 2016 2004, 2009	N	Y	N	6,146 (98)	102 missing coordinates
Lesotho	• (http://www.chal.org.ls/hospitals.php) • (https://www.hfgproject.org/wp-content/uploads/2015/02/Lesotho-Health-Systems-Assessment-2010.pdf)	2016 2016	N	Y	N	117 (92)	9 missing coordinates
Liberia	• (https://data.hdx.rwlabs.org/dataset/health-facilities-liberia-oct-2014) • (https://www.lisgis.net/index.php)	2015 2016	Y	N	N	740 (100)	
Madagascar	• Personal communication	2012	N	N	N	2,677 (99)	13 missing coordinates
Malawi	• (https://data.humdata.org/dataset/malawi-health) • (http://www.cham.org.mw/uploads/7/3/0/8/73088105/cham_health_facilities_-_1_june_2016.pdf) • Personal communication	2017 2016 2013	Y	N	N	648 (99)	9 missing coordinates
Mali	• (https://data.humdata.org/dataset/mali-healthsites) • (https://data.humdata.org/dataset/mali-healthsites/resource/e152bb47-e14c-471c-b47d-0cc93da531db) • (http://www.washclustermali.org/sites/default/files/micro-coordination_wash_dans_cscoms_et_csrefs_19-02-2013.xls)	2017 2016 2017	Y	N	N	1478 (98)	30 missing coordinates
Mauritania	• (http://cartesanitairemauritanie.blogspot.com/)	2017	N	N	Y	645 (100)	50 missing facility names
Mauritius	• (http://health.govmu.org/English/Pages/default.aspx)	2018	N	N	Y	166 (100)	
Mozambique	• (http://sis-ma.in/?page_id=740) • Personal communication	2017 2013	Y	N	N	1,579 (100)	
Namibia	• (https://mfl.mhss.gov.na/location-manager/locations)	2018	Y	N	N	369 (97)	10 clinics missing coordinates
Niger	• (http://www.who.int/hac/crises/ner/maps/ne_poster.jpg?ua=1) • Personal communication	2017 2013	N	N	Y	2,886 (98)	46 missing coordinates
Nigeria	• (https://data.humdata.org/dataset/nigeria-healthsites) • Personal communication	2016 2012	Y	N	N	20,807 (95)	1,114 missing coordinates
Rwanda	• (https://hmis.moh.gov.rw/) • Personal communication	2017 2014	Y	N	N	572 (100)	
São Tomé & Príncipe	• (https://repositorio.hff.min-saude.pt/bitstream/10400.10/440/1/Apresentacao%201.pdf)	2016	N	N	Y	50 (100)	Facility names missing except for hospitals
Senegal	• (http://www.sante.gouv.sn/ckfinder/userfiles/files/cartesanonze.pdf) • Personal communication	2017 2015	N	Y	N	1,347 (93)	91 missing coordinates
Seychelles	• (https://www.seychellespromo.com/guide/Medical)	2018	N	Y	N	18 (100)	
Sierra Leone	• (https://data.humdata.org/dataset/health-facilities-in-guinea-liberia-mali-and-sierra-leone/resource/816718bb-4929-49f0-854d-85b24a0bdb69)	2017	Y	N	N	1,120 (100)	
Somalia	• (https://data.humdata.org/dataset/somalia-health-facilities)(https://reliefweb.int/map/somalia/somalia-functioning-health-facilities-april-june-2013) • UNICEF & WHO report^45^	2016 2013 2013	Y	N	N	879 (96)	39 missing coordinates
South Africa	• (https://dd.dhmis.org/orgunits.html?file=NIDS%20Integrated&source=nids) • Personal communication	2017 2017	Y	N	N	4,303 (99)	15 missing coordinates
South Sudan	• (https://data.humdata.org/dataset/south-sudan-health)	2017	Y	N	N	1,747 (99)	13 missing coordinates
Sudan	• Personal communication	2016	N	N	N	272 (96)	Only hospitals data available; 10 missing coordinates
Tanzania (mainland)	• (http://hfrportal.ehealth.go.tz/index.php?r=facilities/facilitiesList)	2018	Y	N	N	6,304 (100)	
Togo	• (www.stat-togo.org/nada/index.php/catalog/29/download/990) • (https://www.feuvert-dev.org/uploads/files/Rapport%20de%20mission%20Coordinatrice%20AlterSant_%20-%20partenaires.pdf)	2017 2017	N	Y	N	207 (85)	31 missing coordinates
Uganda	• (http://library.health.go.ug/publications/health-infrastructure-physical-infrastructure/health-facility-inventory) • Personal communication	2018 2017	N	Y	N	3,792 (97)	115 missing coordinates
Zambia	• (http://www.moh.gov.zm/docs/facilities.pdf) • Personal communication	2017 2012	N	Y	N	1,263 (99)	4 missing coordinates
Zanzibar	• (https://www.google.com/url?sa=t&rct=j&q=&esrc=s&source=web&cd=1&cad=rja&uact=8&ved=2ahUKEwjNrNDQ7cXiAhURzoUKHRsRCm8QFjAAegQIBBAC&url=http%3A%2F%2Ftanzania.um.dk%2F~%2Fmedia%2FTanzania%2FDocuments%2FHealth%2FZanzibar%2520Strategic%2520Plan%252014%2520-%252018.pdf%3Fla%3Den&usg=AOvVaw3VMLPmQGTMK3Ftm02R_NqP) • Personal communication	2013 2013	N	N	Y	145 (100)	
Zimbabwe	• (https://data.humdata.org/dataset/zimbabwe-health) • (http://www.tarsc.org/publications/documents/REBUILD%20Gap%20Analysis%20Survey%20Rep%20Final%20Nov%202014.pdf)	2016 2017 2017	Y	N	N	1,236 (97)	35 missing coordinates

Note: Abbreviations MFL - Master Facility List; INGO - Inter-Governmental Organization; WHO - World Health Organization; UNOCHA HDX- United Nations Office for the Coordination of Humanitarian Affairs Humanitarian Data Exchange; HMIS - Health Management Information System; UNICEF - United Nations International Children’s Emergency Fund.

**Online-only Table 2:** Country-specific current Health Sector Strategic Plans (HSSPs) used to validate the number of health facilities in each country, while health sector reports, including the HSSPs, were used to validate the distribution of the health facility types across the health service delivery levels.

Country (Sources of database)	Services at healthcare delivery levels	Database	HSSP No. of facilities
Angola (http://extwprlegs1.fao.org/docs/pdf/ang169620.pdf) (https://www.pmi.gov/docs/default-source/default-document-library/malaria-operational-plans/fy17/fy-2017-angola-malaria-operational-plan.pdf?sfvrsn=7)	I. Primary/municipal: **District/Municipal Hospitals** and **Local Health Centres** offer emergency, outpatient, antenatal, nursing, maternity, EPI, surveillance and testing of HIV and support. **Health Post I** and **Health Post II** facilities are first point of contact for the health system and provide basic outpatient services	• 1152 Posto de Saude • 3 Centro Sanatorio Materno Infantil • 39 Centro Materno Infantil • 231 Centro de Saude • 100 Municipal Hospitals • 32 Hospitals	• 1505 Health Posts • 332 Health Centers • 40 Maternal-Infant Centers • 155 Municipal Hospitals
	I. Secondary/provincial: **Provincial/General Hospitals** offer specialized services and receive patients referred from primary level facilities	• 11 Provincial hospitals • 3 General Hospitals • 1 Regional Hospital	NR
	II. Tertiary/central: **Teaching Hospitals** and **National Specialized Hospitals** are the highest referral level and offer specialized care and training of medical professionals	• 3 Central Hospitals	• 3 Central Hospitals
Benin (https://www.urc-chs.com/sites/default/files/Benin_Pilot_Test_Assessment_Report.pdf) (https://vaccineamc.org/country/benin/documents/)	I. Peripheral: First level services offering curative, preventive, promotional and rehabilitation services. **Village Health Units** are first point of care; the second level is the health centre categorized as either **Commune Health Centre** or **Arrondissement Health Centre**. **The Zonal Hospital** is the first referral level for specialist care	• 9 Unites de sante de village • 4 Clinique • 132 Centre Communal de Santé • 27 Dispensaire • 306 Centre de Santé d’Arrondissement • 8 Centre de Santé de Sous-Prefecture • 3 Centre Médico-Social • 24 Centre Medical • 1 Centro de Sante de Circonscription Urbaine • 246 Centre de Santé • 11 Centre de Santé Central • 20 Other Hôpitals • 21 Hôpitals de Zone	• 425 Centre de Santé d’Arrondissement • 75 Centre de Santé de Commune • 27 Hôpitals de Zone
	II. Intermediate: **Departmental Referral Hospitals** are the second referral facilities	• 5 Centre Hospitalier Départemental	• 5 Centre Hospitalier Départemental
	III. Central: The **National University Hospital Centre (CNHU)** is the national referral facility, providing highly specialized services and serves as a training centre	• 1 Centre National Hospitalier Universitaire	• 1 Centre National Hospitalier Universitaire
Botswana (https://www.moh.gov.bw/clinics.html)	I. Primary: **Health posts and mobile stops** are first point of care to the national system and offer mainly outpatient care. Mobile stops are operated by clinics, do not have permanent structure and mainly offer promotive and preventive services. **Clinics** offer primary health care and outpatient services including general consultations, treatment of injuries and minor illnesses	• 331 Health Posts • 264 Clinics	NR
	II. Secondary: **Primary Hospitals** are general hospitals that are equipped to deal with most diseases, injuries & immediate threats to health. **District Hospitals** in addition to primary care, offer intensive and pediatric care; Emergency services, surgery and intensive care; x-ray and diagnosis, dental care services; eye care services, orthopedic services	• 17 Primary Hospitals • 10 District Hospitals	• 16 Primary Hospitals • 12 District Hospitals
	III. Tertiary: **Referral Hospitals** are equipped to handle complex diseases referred from lower level facilities	• 2 Referral Hospitals	
Burkina Faso^46^ (http://origin.who.int/pmnch/topics/part_publications/pmnch2012report_burkina_faso_case_study_0920.pdf) (https://www.afro.who.int/sites/default/files/2018-08/Profil%20sanitaire%20du%20Burkina%20%202.pdf)	I. Primary (level 1): first point of entry to the health system. **Health posts** provide general out-patient care for infectious diseases and minor injuries performed by voluntary CHWs. **Community health and welfare promotion centres (CSPS)** provide promotional, preventive, and curative care, antenatal care services, out-patient management, EPI, counseling, family planning, and social mobilization	• 81 Dispensaries • 1537 Community Health and Welfare Promotion Centres	NR
	II. Primary (level 2): **Centre Médical (CM)** and **Centre Médical avec Antenne Chirurgicale (CMA)** offer first level referral care and surgical services	• 41 Medical Centres (CM) • 49 **Centre Médical avec Antenne Chirurgicale** (CMA)	• 42 **Centre Médical avec Antenne Chirurgicale** (CMA)
	III. Secondary (level 3): **Regional Hospitals** offer complex services referred from primary hospitals	• 9 Regional Hospitals	• 6 Regional Hospitals
	IV. Tertiary (level 4): **National and Teaching Hospitals** provide all services at lower levels in addition to offering training and research facilities	• 3 University Hospital Centres • 1 National Hospital	• 1 National Hospital
Burundi^47^ (http://www.aho.afro.who.int/profiles_information/index.php/Burundi:Service_delivery_-_The_Health_System)	I. Peripheral: **Health Centres** offers a minimum package of services, including treatment and prevention, consultation, laboratory, pharmacy, health promotion and health education services, and in-patient observation. **District Hospitals** are first referral facilities.	• 616 Health Centres • 46 District Hospitals	• 897 Health Centres • 69 Hospitals^a^
	II. Intermediate: **Regional Hospitals** offer specialized psychiatric services, including medication and psycho-education	NR	
	III. Central: **Specialized Hospitals**, **National Hospitals** and the **University Hospital** are the highest referral centres and provide highly specialized services and referrals from all other facilities countrywide	• 3 Tertiary Hospitals	
Cameroon^48^ (http://www.nationalplanningcycles.org/sites/default/files/planning_cycle_repository/cameroon/cameroon_-_sss_validee_par_le_ccss_5_janvier.pdf) (http://www.statistics-cameroon.org/downloads/pets/2/Rapport_principal_Sante_anglais.pdf)	I. Peripheral: **Sub-divisional Medical Centres** (CMA), **Health Centres** (CS & CSI) and **Dispensaries** offer minimum services including preventive and curative services and health promotion activities. Serious cases are referred to **District Hospitals** which are the first point of referral care	• 223 Centre Medical d’Arrondissement • 362 Centre de Sante • 2241 Centre de Sante Integre • 48 Dispensaire • 3 Clinics • 165 District Hospitals	• 3786 Centre de Sante Integre & Centre Medical d’Arrondissement • 218 District Hospitals
	II. Intermediate: **Regional Hospitals** and **General Hospitals** offer specialized healthcare services including inpatient care	• 14 Regional Hospitals • 3 General Hospitals	• 15 Regional Hospitals
	III. Central: **General Referral Hospitals**, **University Hospital Centres** and **Central Hospitals** offers surgical care, obstetrics, gynaecology, radiology, intensive care, emergency and outpatient services. They serve as research and surveillance centres	• 2 Central Hospitals	• 15 General & Central Hospitals
Cape Verde (https://www.insp.gov.cv/index.php/documentos/outors-documentos/36-plano-nacional-de-desenvolvimento-sanitario-2012-2016-volume-ii/file)	I. Primary/municipal: Basic Health Units (**Unidades Sanitárias de Base (USB)**, Health Posts **(Postos de Saúde (PS)** and Health Centres **(Centros de Saúde (CS)** are all first point of care and provde basic preventive and curative services	• 26 Health Posts • 31 Health Centres	• 113 Basic Health Units • 34 Health Posts • 30 Health Centres • 1 Polyclinic
	II. Secondary/regional: **Regional Hospitals** (Hospitais Regionais (HR) provide care for cases of lesser complexity referred from health centres	• 7 Regional Hospitals	• 3 Regional Hospitals
	III. Tertiary/central: **National Reference Hospitals**/**Central Hospitals** (Hospitais Centrais (HC) provides highly specialized care for complex medical cases referred from lower health facilities	• 2 Central Hospitals	• 2 Central Hospitals
Central African Republic (https://www.afro.who.int/sites/default/files/2017-07/RCA%20Rapport%20de%20l%27enqu%C3%AAte%20HeRAMS%20%202016%20Version%20finale_0.pdf) (https://www.ghdonline.org/uploads/Health_Provision_in_the_Central_African_Republic_latest_draft.pdf) Ministère de la Santé Publique et de la Population [Central African Republic]. Politique Nationale De Lutte Contre La Lepre. Bangui, Julliet 2007. (2007) - *Document no longer vailable online*.	I. Peripheral: **Prefectural/District Hospitals**, **Health Centre** and **Health Posts** are first point of care providing promotional, preventive, and curative services	• 326 Poste de santé • 44 Centre de Santé “A” • 45 Centre de Santé “B” • 57 Centre de Santé “C” • 2 Centre de Santé “D” • 61 Centre de Santé “E” • 12 Prefectural Hospitals	• 445 Health Posts • 31 Health Centres “A” • 22 Health Centres “B” • 104 Health Centres “C” • 11 Health Centres “D” • 13 Health Centres “E” • 13 Prefectural Hospitals
	II. Regional: **Regional University Hospitals** are stand‐alone facilities that offer specialised services for patients referred from prefectural hospitals	• 5 Regional University Hospitals	• 5 Regional Hospitals
	III. Central: **Central Hospitals** (Hôpital Centraux) are the highest referral facilities and offer specialized care including training	• 3 Central Hospitals	• 4 Central Hospitals
Chad^49^ (http://www.nationalplanningcycles.org/sites/default/files/country_docs/Chad/plan_national_de_developpement_sanitaire_ii_2013-2015.pdf)	I. Peripheral: **Health Centres** are the lowest level of primary health service. They are the first point of contact with health service and provide basic and primary health care	• 1205 Health Centres	• 1028 Health Centres
	II. Districts: **District Hospitals** are first level referral hospitals equipped to handle special cases including maternal and child health	• 70 District Hospitals	• 72 District Hospitals
	III. Regional: **National Referral Hospitals** are tertiary level hospitals which are well equipped to offer specialized care and tertiary education	• 7 Regional Hospitals • 1 National Hospital	• 8 Regional Hospitals • 1 National Hospital
Comoros (http://www.nationalplanningcycles.org/sites/default/files/country_docs/Comoros/pnds_05_mai_2010_documentvf_en.pdf)	I. Health District: **District Health Centres** and **Health Posts (HP)** offer primary care to the community	• 1 Dispensaire • 45 Poste de Santé • 12 Centre de Santé • 3 Centre Médico-Urbain • 2 Centre Médico-Chirurgical	• 4 NGO facilities • 52 Health Posts • 3 Centre Médico-Urbain • 2 Centre Médico-Chirurgical • 12 District Health Centres
	II. Island/regional: Each island has a **Regional Hospital (CHR**) except the island of Ngazidja where the reference is the National Hospital	• 2 Regional Hospitals	• 2 Regional Hospitals
	III. Central: **National Hospital (CHN)** serve as highest referral facilities for specialized care	• 1 National Hospital	• 1 National Hospital
Congo (http://www.nationalplanningcycles.org/sites/default/files/country_docs/Congo/ppac_2012-2016_congo.pdf)	I. Peripheral**: Hôpitaux de base**, **Centre de santé intégré (CSI)**, Centre de Traitement Ambulatoire (CTA), Centre de Dépistage et de Traitement (CDT) and other specialized treatment centres	• 302 Centre de Santé Intégré • 10 Base Hospitals • 9 Comboutique Hospitals • 2 Hospitals	• 12 Clinics • 16 Base Hospitals
	II. Intermediate: Hôpitaux Généraux	• 4 General Hospitals	• 5 General Hospitals
	III. Central: Specialist Facilities and Centre Hospitalier et Universitaire (CHU)	• 1 University Hospital Centre	• 1 University Hospital
Cote d’Ivoire^50^ (http://www.nationalplanningcycles.org/sites/default/files/planning_cycle_repository/cote_divoire/pnds_2016-2020.pdf) (http://www.nationalplanningcycles.org/sites/default/files/country_docs/Cote%20DIvoire/psn_2011-2015_vih_sida.pdf)	I. Peripheral/Operational: **Rural Health Centres (CSR)** are the first point of contact offering basic out-patient, promotional, preventive and maternity services. **Urban Health Centres (CSU)** offer all services by CSRs in addition to specialized treatment such as for HIV and TB. **General Hospitals a**re the first referral centres	• 1330 Rural Health Centres • 329 Urban Health Centres • 33 Medical Centres • 77 General Hospitals	• 1237 Rural Health Centres • 514 Urban Health Centres • 66 General Hospitals
	II. Intermediate/Regional: **Regional Hospitals** and Specialized Hospitals receive referrals from general hospitals and offer services such as general medicine, pediatrics and obstetrics	• 19 Regional Hospitals	• 17 Regional Hospitals
	III. Central: **Teaching Hospitals (CHU**) & National specialized care institutes offer medical training and national referral services	• 4 University Hospitals	• 4 University Hospitals
DRC (https://www.pmi.gov/docs/default-source/default-document-library/malaria-operational-plans/fy-2018/fy-2018-democratic-republic-of-the-congo-malaria-operational-plan.pdf?sfvrsn=5) (https://www.medbox.org/national-policy-project-fighting-to- malaria- download.pdf)	I. Peripheral/health zone: Government **Health Centres (HC)** provide primary care services. **General Referral Hospitals** are the first level of referral services	• 177 Clinique • 132 Dispensaire • 4231 Poste de Santé • 8,512 Centre de Santé • 160 Centre de Santé de Référence • 4 Centre de Santé Municipal • 98 Polyclinique • 834 Centre Médical • 3 Centre Medico-Chirurgical • 5 Hôpital Secondaire • 370 Hôpital Général de Référence	• 8266 Health Centres • 393 General Referral Hospitals
	II. Intermediate/provincial: **Provincial Hospitals** are the secondary referral facilities	• 302 Hôpital • 72 Centre Hôpitalier	• 6 Provincial Hospitals
	III. Central: **National Hospitals** and **Reference Centres** offer specialized services and training	• 3 Hospitalier Universitaire	• 3 University Hospitals
Djibouti (http://www.nationalplanningcycles.org/sites/default/files/country_docs/Djibouti/pnds_2013_2017_partie_2_version_du_80113.pdf) (https://www.ilo.org/dyn/natlex/docs/ELECTRONIC/76658/80917/F1028108119/DJI-76658.pdf)	I. Primary**: Health post**s and **Community Health Centres** provide basic curative and preventive care, local health promotion & education & ensures community health support. Health Centres supervise the health posts and refer complex cases to **District Hospitals**. **Intermediate Health Centres** offer both outpatient and inpatient care	• 41 Health Posts • 12 Community Health Centres	NR
	II. Secondary: The **Regional Hospital** is a second-level health facility. It supports referrals from health posts and Intermediate Health Centres. It offers all services provided by level 1 and additional health services such: medicine, pediatrics, routine surgery, obstetrics and gynecology, emergency and ambulatory services, dental and ophthalmic care	• 5 Hospital Medical Centres	• 5 Medical Centres (District & Regional Hospitals)
	III. Tertiary: **National Referral Hospitals** and **Special Facilities**. They have a national scope, provide highly skilled care and undertake research and training	• 8 Tertiary Hospitals	• 4 Tertiary Hospitals
Equatorial Guinea (http://www.aho.afro.who.int/profiles_information/index.php/Equatorial_Guinea:Index) Ministere de la Sante et du Bienêtre Social [Equatorial Guinea]. Initiative Faire Reculer le Paludisme Plan Strategique 2009-2013. (2008) - *Document no longer vailable online*.	I. Peripheral/operational: **Health Posts and Health Centres** are first point of care and provide basic routine outpatient services. **District Hospitals provide** more comprehensive care for referral**s from** health posts and health centres	• 29 Health Centres • 11 District Hospitals	• 161 health posts • 42 health centres^b^
	II. Intermediate/regional: **Provincial Hospitals**	• 5 Provincial Hospitals	• 5 Provincial Hospitals
	III. Central: Regional Hospitals	• 2 Regional Hospitals	• 2 Regional Hospitals
Eritrea (http://documents.worldbank.org/curated/en/461991468770937666/pdf/297930PAPER000182131587616.pdf) National Malaria Control Program [Eritrea]. Malaria Five-Year Strategic Plan (2015-2019). Ministry of Health, Department of Public Health Communicable Diseases Control Division. (2014) - *Document no longer vailable online*.	I. Primary: **Community-based health services and Health Stations** provide Basic Health Care Package (BHCP) based services by empowering communities, mobilizing and maximizing resources. **Community Hospital** is the referral facility for the primary health care level in addition to primary care, they offer obstetric and general surgical services	• 2 Mini Clinic • 6 Clinics • 186 Health Stations • 53 Health Centres • 5 Mini Hospitals	• 184 Health Stations • 52 Health Centres
	II. Secondary: **Regional (Zoba) Referral Hospitals and Sub-Zoba Hospitals** serve as referral facilities for the lower level facilities, act as training institutions and provide facilities for research. Provide general surgery, deliveries, laboratory, ophthalmic care, radiology, dental, obstetric, and gynecological services	• 16 Hospitals	• 13 Community Hospitals • 6 Zonal Referral Hospitals
	III. Tertiary: **National Referral Hospitals**. Serves as centre of excellence for specialised training/education and research	• 1 National Referral Hospital	• 5 National Referral Hospitals
Ethiopia (https://www.globalfinancingfacility.org/sites/gff_new/files/Ethiopia-health-system-transformation-plan.pdf)	I. District: satellite **Health Posts** and **Health Centres** provide primary care services. Health centres also serve as a first curative referral centre for Health Posts. **Primary Hospitals** offer referral care from health posts and health centres	• 3135 Clinics • 1002 Health Posts • 769 Health Centres • 10 Nucleas Health Centres • 138 Health Stations • 144 Hospitals • 5 District Hospitals	• 16,440 Health Posts • 3,547 Health Centres
	II. Regional: **General Hospitals** provides training, inpatient and ambulatory services and serves as a referral Centre for primary hospitals.	• 3 Zonal Hospitals • 4 General Hospitals	• 311 Hospitals^c^
	III. National: **Referral & Specialized Hospitals** Handle more complicated and sophisticated health care, including the clinical management of non-communicable diseases.	• 4 Referral Hospitals • 1 National Hospital	
Gabon (http://csgabon.info/file/f2/Plan%20National%20de%20Developpement%20Sanitaire%20du%20Gabon%202011-2015.pdf)	I. Operational: **Health Centres**, **Dispensaries** and **Health Posts** are first level of the health pyramid and offers first point of care including primary care. **Medical Centres** serve as the reference level for lower level facilities.	• 387 Dispensaires • 92 Centres de Santé • 4 Centres de Santé Urbain • 43 Centres Médical	• 157 Health Huts • 413 Dispensaries • 34 Health Centers • 47 Departmental Hospitals
	II. Intermediate/technical support: **Regional Hospitals** are referral facilities for the operational level and offer research operations and training	• 1 Centre Hospitalier Urbain • 10 Hospitalier Régional	• 9 Regional Hospitals
	III. Central/strategic: **National**, **Psychiatric & Specialized Hospitals** – highest referral facilities at national level and offer specialized care	• 3 Hôpital Coopération • 2 Centre Hospitalier Universitaire	• 4 University Hospitals
Gambia (https://www.mindbank.info/item/5970)	I. Clinics and Minor Health Centres: are often the first point of care and includes treatment of minor illnesses and referrals, environmental health & sanitation, antenatal, delivery and postpartum care, home visits & community health promotion	• 46 Clinics	• 634 Primary Health Villages • 40 Community Clinics
	II. Secondary: **Minor Health Centres** provide Reproductive and Child Health (RCH) services, nutrition services, control of common endemic diseases, Family Planning (FP) services, Health promotion and protection and provision of essential drugs and vaccines. **Major Health Centres** serve as referral points for minor health Centres in addition to offering minor surgeries, radiology and laboratory services	• 44 Health Centres (minor) • 7 Health Centres (major)	• 41 Minor Health Centres • 6 Major Health Centres
	III. Tertiary: **Regional Hospitals** offer specialist care and referral services including dental and eye care services, laboratory and radiology services. **Teaching Hospitals** offer all services provided at regional hospital level, specialist hospital, services, and mortuary services	• 3 Hospitals • 2 General Hospitals • 1 Teaching Hospital	• 7 Tertiary Hospitals
Ghana^51^ (https://www.google.com/url?sa=t&rct=j&q=&esrc=s&source=web&cd=1&cad=rja&uact=8&ved=2ahUKEwjyvqfPt8_iAhVJURoKHfehAV8QFjAAegQIAxAC&url=http%3A%2F%2Fapps.who.int%2Fhealthinfo%2Fsystems%2Fdatacatalog%2Findex.php%2Fcatalog%2F21%2Fdownload%2F88&usg=AOvVaw2fz26L9PKg_VQty4Tfx-BP) (https://www.ecoi.net/en/file/local/1093121/90_1236873017_accord-health-care-in-ghana-20090312.pdf) (https://www.ghanahealthservice.org/downloads/FACTS+FIGURES_2017.pdf)	I. Community: **Village** or **Community Health Posts** provide preventive and primary health care services.	• 632 Community-based Health Planning and Services (CHPS)	• 4185 Community-based Health Planning and Services (CHPS) zones
	II. Sub District: **Health Centres** and **Clinics** provide basic curative care, disease prevention and maternity services	• 398 clinics • 740 Health Centres • 12 polyclinics	• 1003 Clinics • 855 Health Centres • 34 Polyclinics
	III. District: **District Hospitals** provide support to sub-districts in disease prevention and control, health promotion, referral outpatient and inpatient care, training and supervision, maternity services, management of complications, emergency services, and surgical care.	• 77 Hospitals • 81 District Hospitals • 6 Municipal Hospitals	• 137 District Hospitals • 267 Hospitals
	IV. Regional: **Regional Hospitals** and **polyclinics** provide specialized clinical and diagnostic care, management of high-risk pregnancies and complications of pregnancy, technical and logistical back up for epidemiological surveillance, medical research and training of medical personnel. The **polyclinics** also serve as first point of contact in urban centres and therefore provide a mixture of preventive and curative care and use the regional hospitals for referrals	• 3 General Hospitals • 8 Regional Hospitals	
	V. Tertiary: **Teaching Hospitals** offer specialized clinical and maternity services, undertake research, and provide highest level of undergraduate and postgraduate training in health and allied areas.	• 3 Teaching Hospitals	
Guinea (http://www.nationalplanningcycles.org/sites/default/files/country_docs/Guinea/plan_national_developpement_sanitaire_2015-2024_guinee_fin.pdf) (https://www.pmi.gov/docs/default-source/default-document-library/malaria-operational-plans/fy-2018/fy-2018-guinea-malaria-operational-plan.pdf?sfvrsn=5)	I. Primary/health district: **Health Posts** provide basic primary care. **Health Centres** provide preventive and curative care and supervise the health posts. **District Hospitals** serve as a reference for health Centres	• 1241 Poste de Sante • 470 Centre de Sante • 25 Hopital Prefectoral	• 925 Health Posts • 410 Health Centers • 5 Improved Health Centers • 33 Communal Medical Centers & Prefectural Hospitals
	II. Secondary/regional: **Regional Hospitals** serve as a reference for the districts and receive referrals from district hospitals	• 7 Hopital Regional	• 7 Regional Hospitals
	III. Tertiary/national: **University Hospitals** are the highest level of reference for specialized care	• 3 National Hospitals	• 3 National Hospitals
Guinea-Bissau (http://www.nationalplanningcycles.org/sites/default/files/country_docs/Guinea-Bissau/pndsii_2008-2017_gb.pdf)	I. Local/health areas: **Basic Health Units** are supported by CHWs and traditional midwives. **Health Centres** are classified as A, B and C which differentiates them, for example, surgery is provided at health Centres A. Health Centres can also be classified as rural or urban and their coverage area is extended by mobile teams. This level also consists of **Sectoral Hospitals**	• 4 Hospitals	NR
	II. Regional: Regional Hospitals	• 3 Regional Hospitals	• 5 regional hospitals
	III. Central: **National Hospital** (National Simão Mendes Hospital (HNSM)) and **Specialized Reference Centres**	• 1 National Hospital	• 1 National Hospital
Kenya (http://publications.universalhealth2030.org/uploads/kenya_health_policy_2014_to_2030.pdf) (http://e-cavi.com/wp-content/uploads/2014/11/kenya-health-sector-strategic-investiment-plan-2013-to-2017.pdf) (https://www.hfgproject.org/wp-content/uploads/2015/02/Lesotho-Health-Systems-Assessment-2010.pdf)	I. Community level: provide health promotion services, and supply services. In the essential package, all **Non-Facility Based Health** and **Related Services** are classified as community services – not only the interventions provided through the Community Health Strategy as defined in NHSSP II		
	II. **Dispensaries** are at the lowest level of the public health system and are the first point of contact with patients.	• 348 Clinics • 4352 Dispensaries	• 169 Clinics • 3715 Dispensaries
	III. **Health Centres** offer basic curative and preventive services, reproductive health, minor surgical services, outreach services,	• 1044 Health Centres	• 872 Health Centres
	IV. Primary referral facilities: **District Hospitals** receive referrals from primary care facilities and offer inpatient services, surgery and obstetrics.	• 19 Hospitals • 89 Mission Hospitals • 158 Sub-District Hospitals • 121 District Hospitals	• 347 County Hospitals
	V. Secondary referral facilities: **Provincial Hospitals** Offer specialized services such as pediatrics, surgery and serve as teaching centres given the availability of specialized staff.	• 3 County Referral Hospitals • 10 Provincial General Hospitals	• 7 Provincial General Hospitals
	VI. Tertiary referral facilities: **National Teaching and Referral Hospitals** are the referral centres of excellence and providing complex health services requiring more complex technology and highly skilled personnel	• 2 National Referral Hospitals	• 2 National Referral Hospitals
Lesotho (https://www.hfgproject.org/wp-content/uploads/2015/02/Lesotho-Health-Systems-Assessment-2010.pdf)) (http://www.nationalplanningcycles.org/sites/default/files/country_docs/Lesotho/19_04_2013_lesotho_hssp.pdf)	I. Community/primary: **Health Posts** provide community outreach services, promotive, preventive, and rehabilitative care in addition to organizing health education gatherings and immunization efforts. **Health Centres** are the first point of care within the formal health system and offer immunizations, family planning, and postnatal and antenatal care on an outpatient basis. They also supervise community public health efforts and training volunteer CHWs	• 95 Health Centres	• 188 Health Centres,
	II. District/secondary: **Filter Clinics** function as “mini-hospitals,” offering curative and preventive services, limited inpatient care. laboratory and radiology services. **District Hospitals** handle more complex cases including minor and major operative services, ophthalmic care, counseling and care of rape victims, radiology, dental services, mental health services, and blood transfusions and preventive care, TB, HIV, and noncommunicable diseases	• 8 Mission Hospitals • 11 District Hospitals • 2 Filter Clinics	• 18 Hospitals • 3 Filter Clinics
	III. National/tertiary: **National Referral Hospitals** receive referrals from filter clinics and district hospitals	• 1 National Referral Centre	• 1 Referral Hospital
Liberia (https://washwatch.org/uploads/filer_public/e3/d9/e3d9b04d-5f6e-4414-b223-badc67abadf7/national_health_and_social_welfare_policy_and_plan_liberia_2011-2021.pdf)	I. Primary: **Community-based services** occur within the radius of a Public Health Care (PHC) facility catchment population. **Non-facility-based service delivery points** (SDPs) (e.g. mobile clinics or community-based providers) offer services by a skilled provider on a regular basis outside of a health facility. **Clinics** offer the whole Essential Package of Health Services (EPHS) for the primary level and may or may not have a laboratory	• 661 Clinics	• 496 Clinics
	II. Secondary: **Health Centres** offer 24-hour primary care services complemented by a small laboratory and inpatient services. **County Hospitals** are the most comprehensive type of referral facility for the counties and serve as the referral facilities for the clinics and health centres	• 41 Health Centres • 8 Mission Hospitals • 29 Hospitals	• 43 Health Centres • 27 Hospitals
	III. Tertiary: **John Fitzgerald Kennedy Medical Centre (JFKMC)**, the national referral hospital is the top teaching hospital for physicians and medical doctors. There are also a limited number of county hospitals serving as **Regional Referral Hospitals** which are located within reasonable access of the county hospitals that refer to them and provide specialized consultative care such as orthopedics and ear, nose and throat services.	• 1 National Referral Hospital	• 1 National Referral Hospital
Madagascar (http://www.nationalplanningcycles.org/sites/default/files/planning_cycle_repository/madagascar/pdss_2015.pdf) (https://www.pmi.gov/docs/default-source/default-document-library/malaria-operational-plans/fy16/fy-2016-madagascar-malaria-operational-plan.pdf?sfvrsn=5)	I. Primary: first point of contact in the system. **Basic Health Centres I (CSB I)** provide curative and preventive care and are run by para-medical officers. **Basic Health Centres II** (**CSB II**) provide the same healthcare only that urban dispensaries and maternity care Centres are attached to them, and they are run by a Physician	• 910 Health Posts • 1642 Health Centres	• 956 Basic Health Centres I • 1632 Basic Health Centres II
	II. Secondary: **District Hospital Centres I** are based in district headquarters but offer similar services to those offered in a CSB II. **District Hospital Centres I**I also based in district headquarters but function as District Hospital Centres I and additionally offer emergency surgery and comprehensive obstetrical care and referral health services	• 125 hospitals	• 87 First-Referral Hospitals
	III. National: **Referral hospitals (CHR)** and **University Hospitals (CHU**) serve as specialized referral Centres and the **Regional Hospitals** serve patients requiring a higher level of care that serve as tertiary care health facilities		• 16 Regional Referral Hospitals • 22 University Hospitals
Malawi (http://www.nationalplanningcycles.org/sites/default/files/country_docs/Malawi/2_malawi_hssp_2011_-2016_final_document_1.pdf) (https://dhsprogram.com/pubs/pdf/SPA20/SPA20%5BOct-7-2015%5D.pdf)	I. Primary: Community Initiatives, Health Posts, Dispensaries, Maternity Facilities, Health Centres, and Community/Rural Hospitals. These cadres provide a range of mostly promotive and preventable services and some curative services	• 22 Clinics • 87 Health Post/Dispensaries • 457 Health Centres • 2 Community Hospitals • 25 Rural Hospitals	• 28 Health Posts • 88 Clinics • 48 Dispensaries • 477 Health Centres • 97 Hospitals^d^
	II. Secondary: **District Hospitals** are referral facilities for the primary level of care and provide both inpatient and outpatient services for their target populations	• 27 Mission Hospitals • 24 District Hospitals	
	III. Tertiary: **Central Hospitals** act as referral facilities for the district hospitals while providing services in their regions. Central hospitals also have the mandate to offer professional training, conduct research, and provide support to the districts	• 4 Central Hospitals	
Mali^52^ (https://www.sida.se/contentassets/5d74d2a4ba184f78aab8d2930dcb5c61/the-national-health-accounts-process-in-mali_625.pdf)	I. Primary/health district: **Community Health Centres** (Centre de Santé Communautaire (CSCom)) provide basic preventive, promotional and curative health services with most having capabilities for maternal and child health	• 94 Clinics • 1294 Community Health Centres	• 1086 Community Health Centres
	II. Regional/intermediate: **Referral Health Centres** (CSRef) and **Polyclinics**. CSRefs offer first level referral care for emergencies, obstetrics, surgical operations, in-patient care.	• 61 Referral Health Centres • 11 Polyclinics	• 60 Reference Health Centres (CSRef)
	III. Tertiary/central: **Referral Hospitals** act as second level of referral and offer all the medical and surgical specialties, high-tech laboratories and medical imaging services	• 12 Regional Hospitals • 3 University Hospitals	• 8 Hospitals • 3 National Hospitals
Mauritania (http://www.nationalplanningcycles.org/sites/default/files/country_docs/Mauritania/plannationaldveloppementsanitaire2012-2020.pdf) (https://www.uhc2030.org/fileadmin/uploads/ihp/Documents/Country_Pages/Mauritania/Analyse%20de%20situation%20secteur%20de%20sant%202011%20VF.pdf) (https://www.google.com/url?sa=t&rct=j&q=&esrc=s&source=web&cd=1&cad=rja&uact=8&ved=2ahUKEwjsmfKsjc_iAhWnxoUKHZTwABoQFjAAegQIAhAC&url=http%3A%2F%2Fapps.who.int%2Fhealthinfo%2Fsystems%2Fdatacatalog%2Findex.php%2Fddibrowser%2F55%2Fdownload%2F168&usg=AOvVaw35u_wxI6WN1s9tr6inSV8J) Ministère de la Santè et des Affaires Sociales [Mauritania]. Plan stratégique national de lutte contre les èpidèmies de paludisme 2006–2010. (2005). – *Document no longer available online* Ministere de la Sante [Mauritania]. Carte Sanitaire Nationale de la Mauritanie. (2014) *Document no longer available online*	I. Moughataa/peripheral: **Basic Health Units** mainly serve to extend service provided at health posts or health Centres. **Health posts** are run by a nurse and offer primary curative consultation, pre-natal consultation, delivery, monitoring of children under 5 years, immunization, birth spacing and distribution of essential drugs. **Health Centres (Category B)** provide primary curative consultation services, pre-natal consultation, delivery, monitoring of children under 5 years, immunization, birth spacing and distribution of essential drugs. **Health Centres (Category A)** provide inpatient services, laboratory, radiology department and a department of dental surgery in addition to services offered at health centers B.	• 552 Health Posts • 75 Health Centres	• 545 Basic Health Units • 530 Health posts • 67 Health Centres
	II. Intermediate: **Moughataa Hospitals**, **Regional Hospitals** and **Regional Medical Centres** offer all basic services in addition to more specialist treatments/procedures	• 14 Hospitals	• 2 Moughataa Hospitals • 6 Regional Hospitals • 6 Regional Medical Centres
	III. Tertiary: national referral and includes **General (National) Hospitals** provide tertiary care in all medical and surgical specialties, including training and research activities. **Specialized Hospitals**, develop high-precision technology in a single specialty in addition to playing the same function of the national hospital	• 4 General Hospitals	• 4 General Hospitals
Mauritius (http://health.govmu.org/English/Documents/2018/ANNUAL%20REPORT%202017%20FOR%20PRINTING.pdf) (http://health.govmu.org/English/Documents/whitepap.pdf)	I. Primary: **Area Health Centres** (AHCs), **Medi-Clinics** (MCs) and **Community Hospitals** (CH) are the first points of contact and services include X-Ray, dental care, laboratory tests and pharmaceutical services for essential drugs not requiring specialist advice. **Community Health Centres** (CHCs) provide health promotion, health education, family planning and primary health care diagnostic and treatment services	• 21 Area Health Centres • 2 Health Centres • 5 Medi-Clinics • 130 Community Health Centres • 2 community hospitals	• 26 Area Health Centres • 2 Medi-Clinics • 3 Family Health Clinics • 125 Community Health Centres • 2 community hospitals
	II. Secondary: **Regional Hospitals** provide accident and emergency services, general medicine, general and specialised surgery, gynaecology and obstetrics, chest medicine, orthopaedics traumatology, paediatrics and intensive care services. Radiotherapy services are provided at Victoria Hospital	• 3 District hospitals • 5 Regional hospitals	• 3 District hospitals • 5 Regional hospitals
	III. Tertiary: National specialized centres e.g. Cardiac Centre		
Mozambique (http://www.nationalplanningcycles.org/sites/default/files/planning_cycle_repository/mozambique/mozambique_-_health_sector_strategic_plan_-_2014-2019.pdf) (http://www.afro.who.int/en/countries.html)	I. Primary: **Health Posts** are staffed by CHWs, elementary nurses and midwifes who provide information, conduct education, communication (IEC) for pregnancy, malaria, WASH, nutrition, routine immunization, nutritional assessments, malaria case management and family planning. **Health Centres I and II** mostly serve rural areas while **Health Centres C and A** mostly serve urban Centres. They all provide essential curative services including vaccination and prevention of local endemic diseases.	• 262 Posto de Saúde • 130 Centro de Saúde Rural I • 982 Centro de Saúde Rural II • 39 Centro de Saúde Urbano A • 56 Centro de Saúde Urbano B • 49 Centro de Saúde Urbano C	• 1395 Level I Facilities
	II. Secondary: **Rural Hospitals**, **District Hospitals** and **General Hospitals** conduct routine surgical interventions with larger diagnostic capacity such as X-ray facilities and serve as first point of reference for level I and II facilities	• 29 Hospital Rural • 16 Hospital Distrital • 5 Hospital Geral	• 48 Level II Facilities
	III. Tertiary: **Provincial Hospitals** provide limited tertiary care including referral for lower level facilities in addition to the primary care services.	• 8 Hospital Provincial	• 7 Level III Facilities
	IV. Quaternary: **Central Hospitals** and **Specialized Hospitals** are in each region and provide a broader range of specialized, curative, surgical and rehabilitative services	• 3 Hospital Central	• 3 Level IV Facilities
Namibia (http://www.nationalplanningcycles.org/sites/default/files/country_docs/Namibia/namibia_national_health_policy_framework_2010-2020.pdf) (https://dhsprogram.com/pubs/pdf/SPA16/SPA16.pdf) (http://s3.amazonaws.com/zanran_storage/hs2020.org/ContentPages/1032599816.pdf)	I. **Outreach services**: Identifies health needs and refers to the clinic. They also provide home based health care services		• 1150 Outreach Points
	II. Primary: **Health Centres** offer promotive, preventive, curative and rehabilitative and supervisory services to the community based health care providers. Supports clinics and engages in in-service training and operational research. **Clinics** offer promotive, preventative and rehabilitative services and management of outreach services	• 287 Clinics • 43 Health Centres	• 309 Clinics and Health Centres
	III. Secondary: **District Hospitals** are used mostly as first referral facilities for clinics and health centres	• 3 Mission Hospitals • 29 District Hospitals	• 29 District Hospitals
	IV. Tertiary: **National Referral Hospitals** and **Intermediate Hospitals** offer teaching facilities and specialized medical services such as: ambulatory, in-patient, maternity emergency obstetric care, and rehabilitative services. They link up with other national and international health care providers	• 3 Intermediate Hospitals • 1 Central Hospital	• 4 Intermediate and Referral Hospitals
Niger^53^ (https://www.who.int/pmnch/media/events/2014/nig_pds.pdf) (http://www.stat-niger.org/statistique/file/Annuaires_Statistiques/snis/Annuaire_statistiques_2016.pdf)	I. Health district/operational level: **Health Huts** are village level facilities supervised by the Integrated Health Centre and provide health care services in rural areas. **Integrated Health Centres (CSI)** are the first level of primary health service and are further classified based on the population they serve (**CSI I** & **II**). **District Hospitals** provides diagnostic services, outpatient and inpatient care, and emergency obstetrics	• 2009 Health Huts • 836 Integrated Health Centres	• 2160 Health Huts • 829 Integrated Health Centres • 33 District Hospitals
	II. Intermediate/regional: **Regional hospitals** primarily act as referral facilities to lower-level facilities at the health district/operational level	• 40 Hospitals	• 6 Regional Hospitals
	III. Central: **National Hospitals**, **General Reference Hospitals** and **Maternal and Child Hospital** handle major illness such as TB, Leprosy in additional to all services of the lower levels with more specialist treatment and procedures	• 1 University Hospital	• 3 National University Hospitals
Nigeria^54–56^ National Agency for Control of HIV/AIDS [Nigeria]. National Baseline Survey of Primary Health Care Services and Utilization in Nigeria: Survey Report. (2011) *– Report no longer available online*	I. Primary: **Primary Health Care (**PHC) facilities provide first point of contact with the national health system. **Comprehensive Health Centres** (CHC), **Model PHC**, **Primary Health Centres**, **Health Clinics**, **Dispensaries** and **Health Posts** all are first point of care in the health system and provide curative and preventive services	• 3058 Health Posts • 3239 Dispensaries • 4354 clinics • 568 Basic Health Centres • 4639 Primary Health Centres • 3402 Health Centres • 434 Comprehensive Health Centres • 58 Model Primary Health Centres • 108 Model Health Centres • 19 Medical Centres • 10 Polyclinics	NR
	II. Secondary: **District** and **General Hospitals** are referral centres for PHC facilities and provide out-patient and in-patient services, general medical and surgical operations, laboratory, blood bank services, rehabilitation and physiotherapy.	• 141 Hospitals • 19 Rural Hospitals • 149 Cottage Hospitals • 16 District Hospitals • 529 General Hospitals • 12 State Hospitals	NR
	III. Tertiary: **Teaching Hospitals**, **Federal Medical Centres**, **National Laboratories** and other **Specialist Hospitals** that provide care on specific diseases such as orthopedic, eye, psychiatric, maternity and pediatric cases	• 19 Federal Medical Centres • 33 Tertiary Hospitals	NR
Rwanda (http://www.moh.gov.rw/fileadmin/templates/Docs/HSSP_III_FINAL_VERSION.pdf) (https://www.pmi.gov/docs/default-source/default-document-library/malaria-operational-plans/fy-2018/fy-2018-rwanda-malaria-operational-plan.pdf?sfvrsn=6)	I. Community health: **Outreach Services** conducted by CHWs and are supervised administratively by those in charge of social services and technically by those in charge of health Centres		
	II. Peripheral: **Dispensaries** offer primary health care, outpatient, and referral. **Health Posts** undertake outreach activities (i.e., immunization, family planning, growth monitoring, antenatal care)	• 37 Health Posts • 2 Secondary Health Posts	• 44 Health Posts
	III. Intermediate: **District Hospitals** offer inpatient/outpatient services, surgery, laboratory, gynecology, obstetrics, and radiology. **Health Centres** offer prevention activities, primary health care, inpatient, referral, and maternity	• 486 Health Centres • 36 District Hospitals	• 450 Health Centres • 40 District Hospitals
	IV. Tertiary: **National Referral Hospitals** offer inpatient/outpatient services, surgery, laboratory, gynecology, obstetrics, and radiology; specialized services including ophthalmology, dermatology, ear nose and throat, stomatology, and physiotherapy. They also offer training to doctors and pharmacists	• 4 Provincial Hospitals • 3 Referral Hospitals • 4 National Referral Hospitals	• 5 Provincial Hospitals • 4 Referral Hospitals
São Tomé and Príncipe (http://www.nationalplanningcycles.org/sites/default/files/country_docs/Sao%20Tome%20and%20Principe/ppac_version_du_31-05-11.pdf) (https://interlusofona.info/wp-content/uploads/2017/10/saude_para_todos_-_estudo_de_caso.pdf) (https://repositorio.hff.min-saude.pt/bitstream/10400.10/440/1/Apresentacao%201.pdf)	I. District/operational: **Community Health Posts** are informal units managed by CHWs and provide basic care, first aid as well as counseling, vaccination and nutrition and reproductive education to the rural community. **Health Posts** are the basic health units that offer basic nursing care and are managed by a general nurse who visits regularly. **Health Centres** receive referrals from health posts and community health posts and provide outpatient care except for a few which offer some inpatient care	• 13 Community Health Posts • 29 Health Posts • 6 Health Centres	NR
	II. Central: **Central Hospitals** provide both general and specialized treatments to both inpatients and outpatients	• 2 Hospitals	NR
Senegal (https://peoplethatdeliver.org/ptd/sites/default/files/resource_contents_files/S%C3%A9n%C3%A9gal%20Country%20Survey%20EN.pdf) (https://www.itdp.org/wp-content/uploads/2014/07/ITDP-Transport-and-Health-Care-Senegal.pdf)	I. Peripheral/health post: **Health Points/Huts** form the lowest level and are the foundation of the health delivery system. Services offered are promotional, integrated package of maternal and child health, malaria, nutrition and, in many cases, family planning services, basic curative including dressing wounds. **Health Posts** are responsible for health points that are within their jurisdiction and provide preventive and primary curative services, prenatal care, family planning, and health promotion	• 1231 Health Posts	• 971 Health Posts
	II. Intermediate/health centre: **Health Centres** are staffed by 1-2 doctors, and 15 -20 health workers and are responsible for health posts within their area and handle all cases referred by health posts. District health Centres provide first-level referrals and limited hospitalization services with between 10-20 beds	• 87 Health Centres	• 70 Health Centres
	III. Central/hospital: **National Hospitals** and **Regional Hospitals** provide highly specialized referral services and training	• 8 Hospitals • 1 General Hospital • 13 Regional Hospitals • 5 National Hospitals • 2 University Hospitals	• 20 Hospitals
Seychelles (http://www.health.gov.sc/wp-content/uploads/SEYCHELLES-NATIONAL-HEALTH-STRATEGIC-PLAN.pdf)	I. Tier 1. District Health Centres provide clinical and rehabilitative services	• 17 District Health Centres	• 17 District Health Centres
	II. Tier 2: Cottage Hospitals provide specialized services	• 3 Cottage Hospitals	• 3 Cottage Hospitals
	III. Tier 3: Mahe (Victoria) National Referral Hospital is the main referral for all district health centres	• 1 National Referral Hospital	• 1 National Referral Hospital
Sierra Leone^57^ (https://www.unicef.org/emergencies/ebola/files/SL_Health_Facility_Survey_2014Dec3.pdf) (http://www.ministerial-leadership.org/sites/default/files/resources_and_tools/NHSSP%202010-2015.pdf)	I. Primary: **Maternal and Child Health Posts (MCHP)** are the first level of contact for patients in rural settings. They offer antenatal, delivery and postnatal care. **Community Health Post**s are situated in small towns and have similar functions to the MCHP with added curative functions. **Community Health Centres** have preventive, promotive and curative functions and offer inpatient care and laboratory services. They can also handle some complications; grave cases of childhood illness; treatment of complicated cases of malaria and inpatient and outpatient physiotherapy for disability	• 11 Clinics • 619 Maternal and Child Health Posts • 217 Community Health Posts • 25 Health Posts • 206 Community Health Centres • 10 Health Centres	• 66 Clinics • 520 Maternal and Child Health Posts • 176 Community Health Posts • 178 Community Health Centres
	II. District: **District Hospitals** are secondary level facility providing referral support to PHUs. They provide out-patient, inpatient and diagnostic services, management of accidents and emergencies, and technical support to primary facilities	• 12 Mission Hospitals • 19 Hospitals	• 11 Mission Hospitals • 30 Government Hospitals
	III. Tertiary: **Regional National Hospitals** provide tertiary care and a wider range of essential drugs and laboratory services than the lower levels. They are staffed by doctors, including male or female OB/GYNs, surgeon, anaesthetist, and paediatrician; midwives; lab and X ray technicians; pharmacist; and dentist and dental technician	• 1 Referral Hospital	
Somalia Zonal Malaria Control Programmes/Ministry of Health [Somalia, Puntland & Somaliland). National Malaria Strategic Plan 2016-2020. (2016). (www.somalilandmoh.org/wp-content/…/Somali-Malaria-Strategic-Plan-2016-2020.pdf) – *link is no longer accessible*.	I. **Primary Health Units (PHUs):** formed from health posts and staffed by at least one trained Community Health Worker (CHW) and provide basic health prevention and promotion services	• 442 Health Posts	• 269 Primary Health Units
	II. **Health Centres (HCs)** are the key service delivery units providing maternal and child health services, a delivery unit and a six-bed observation unit. **Maternal and Child Health centres** and Outpatient Departments not meeting these criteria will become PHUs	• 334 Maternal & Child Health Centres • 24 Health Centres	• 198 Maternal and Child Health Centres
	III. **Referral Health Centres (RHCs**) and **District Hospitals** provide the next level of services that include comprehensive obstetric care	• 70 Hospitals • 3 District Hospitals	• 26 District Hospitals • 10 Regional Hospitals
	IV. **Referral Hospitals** provide 24-hour uninterrupted services and are staffed by resident doctors and specialists	• 5 Regional Hospitals • 1 Referral Hospital	• 8 Referral Hospitals
South Africa (https://www.gov.za/sites/default/files/gcis_document/201707/40955gon627.pdf)	I. Primary: District services: Primary Health Care (PHC) Services: **Community Clinics** and **Community Health Centres (CHC’s)** and **District Hospitals**	• 3435 Clinics • 205 Satellite Clinics • 34 Health Posts • 8 Community Health Centres/Clinics • 284 Community Health Centres • 9 Community Health Centres (After hours) • 1 Medical Centre • 254 District Hospitals	• 3760 Primary Health Care Facilities (clinics, community health centres & district hospitals)
	II. Secondary: Regional Hospitals	• 47 Regional Hospitals	NR
	III. Tertiary: Provincial Hospitals	• 17 Provincial Tertiary Hospitals	NR
	IV. Quaternary: National and Central Referral Centres	• 9 National Central Hospitals	• 10 Central Hospitals
South Sudan (http://www.nationalplanningcycles.org/sites/default/files/country_docs/South%20Sudan/south_sudan_hsdp_-final_draft_january_2012.pdf)	I. Primary: **Primary Health Care Units (PHCUs)** provide basic, preventive, promotive and curative services	• 1375 Primary Health Care Units	• 792 Primary Health Care Units
	II. Intermediate: **Primary Health Care Centres (PHCCs)** deliver diagnostic laboratory services, maternity and inpatient care in addition to services provided by the PHCUs	• 332 Primary Health Care Centres	• 284 Primary Health Care Centres
	III. Referral: **County Hospitals (CHs)** and **State Hospitals (SH)** provide emergency surgical operations in addition to services provided by PHCCs in addition to training and mentoring of interns	• 28 County Hospitals • 9 State Hospitals	• 27 County Hospitals • 7 State Hospitals
	IV. Tertiary: **Teaching Hospitals (THs)** should provide tertiary care in addition to training of medical professionals and conducting research. Mostly they provide basic services due to lack of equipment and qualified personnel	• 3 Teaching Hospitals	• 3 Teaching Hospitals
Sudan^58^ (http://www.nationalplanningcycles.org/sites/default/files/country_docs/Sudan/sudan_25-year_strategic_plan_for_health.pdf)	I. Primary: **Basic Health Units (BHUs)** are the minimum facility level for and are structured and staffed to deliver the essential package of primary healthcare services. **Health Centres** are the first referral point for BHUs and are supposed to be headed by a physician	NR	NR
	II. Secondary: **Rural Hospitals** are referral facilities for primary healthcare facilities and have inpatient capability	• 125 Hospitals • 3 Type A Hospitals • 7 Type B Hospitals • 7 Type C Hospitals • 123 Type D Hospitals	• 250 Rural Hospitals • 166 other public hospitals
	III. Tertiary: **Teaching Hospitals** and **Specialized Hospitals** offer advanced specialized services and are training hospitals for both undergraduate and postgraduate students	• 1 Referral Hospital • 1 National Hospital • 5 Teaching Hospitals	
eSwatini^59^ (http://www.gov.sz/index.php/ministries-departments/ministry-of-health/clinical-services) (http://www.nationalplanningcycles.org/sites/default/files/planning_cycle_repository/swaziland/swaziland_strategic_plan_2008-2013pdf.pdf) (http://www.gov.sz/index.php?option=com_content&view=article&id=565&Itemid=577)	I. Primary: consists of CHWs, clinics and outreach Services including health promotion in rural areas that focus on prevention of malaria and HIV/AIDS. **Clinics** are the first point of care and offer primary care, vaccination and family planning. **Outreach Clinics** provide community-based care and are owned mostly by FBOs and CBOs with little government support	• 23 Clinics • 78 Clinics without Maternity • 12 Clinics with Maternity	
	II. Secondary: **Health Centres** and **Public Health Units** offer both inpatient and outpatient services and act as referral points for clinics. Services provided include dental, VCT, Prevention of mother to child transmission (PMTCT), obstetric, pediatric, and minor surgery	• 8 Public Health Units • 8 Health Centres	• 149 Clinics and Health Centres (approx.)
	III. Tertiary: **Regional Hospitals**, **Specialized Hospitals** and **The National Referral Hospital** handle complicated surgical procedures and specialized services such as psychiatric treatment, TB, dental, emergency and ambulatory services, occupational health, pediatric, and Mortuary services. Complicated cases are referred to South Africa	• 2 Mission Hospitals • 3 Regional Hospitals • 1 Referral Hospital	• 4 Regional Hospitals • 1 Referral Hospital
Tanzania (Mainland)^60,61^ (http://www.tzdpg.or.tz/fileadmin/documents/dpg_internal/dpg_working_groups_clusters/cluster_2/health/Key_Sector_Documents/Induction_Pack/Final_HSSP_IV_Vs1.0_260815.pdf)	I. **Community-Based Health Care** services conduct health promotion of good health practices and preventive measures through CHWs. **Dispensaries** provide preventive and curative outpatient services and oversee all the village health services. **Rural Health Centres** additionally provide in-patient services, maternity care, laboratory, first referral Centre for dispensaries, and mortuary services, and sometimes surgical services. **District/Council Hospitals** are referral centres and perform general surgical and obstetric operations, offer training to middle and operational level personnel and refer patients to regional hospitals	• 5407 Dispensaries • 676 Health Centres • 20 Other Hospitals • 63 Mission Hospitals • 36 Designated District Hospitals • 67 District Hospitals	• 5819 Dispensaries • 614 Health Centres • 37 Council Designated Hospitals • 63 Council Hospitals
	II. **Regional Referral Hospitals (RRHs)** offer specialized treatment in addition to providing all services offered at district level but at a higher level of expertise. They offer referral services from district hospitals, conduct training of middle and operational level health personnel, conduct operational research of health systems and provide technical skills to lower level facilities in the region	• 26 Regional Referral Hospitals	• 27 Regional Referral Hospitals
	III. **Zonal Hospitals** offer advanced specialist care and are involved in outreach visits to other hospitals in the zone	• 10 Referral Hospitals	• 5 Zonal Hospitals
	IV. **National Hospitals: National Referral and University Teaching Hospital** offers advanced care and acts as training centre for high and middle level medical, paramedical, and nursing care personnel, conducts health research and consultancy on various health and medical issues. It also offers clinical and nursing services	• 1 National Hospital	• 1 National General Hospital
Togo^62^ (http://apps.who.int/medicinedocs/documents/s21005fr/s21005fr.pdf)	I. Peripheral/operational: Prefectural Hospital Centres (**Centre Hospitalier Préfectoral (CHP)**, Medical Centres (**Centre Médicosocial (CMS))** and Peripheral Care Units (**Unité De Soins Périphérique (USP))**. CHPs are first point of care in prefecture capitals since there are no lower level facilities and offer curative and preventive services in maternal and child health, family planning promotion of basic sanitation and hygiene	• 118 Peripheral Care Units • 51 Medical Centres • 30 Prefectural Hospital Centres	• 670 Peripheral Care Units • 35 district hospitals
	II. Intermediate/regional: **Specialized Referral Hospitals** and **Regional Hospital Centre (CHR)**. CHRs offer emergency care and surveillance services and are in each of the six health regions and form the second referral level to the primary level facilities	• 5 Regional Hospital Centres	• 6 Regional Hospital Centres
	III. Central/national: University Hospital Centres **(Centre Hospitalier Universitaire (CHU))** and **National Specialist Hospitals**. CHUs are the highest referral centres and undertake training of health professionals and conduct research for the national healthcare system	• 3 University Hospital Centres	• 3 University Hospital Centres
Uganda (https://www.who.int/healthinfo/systems/SARA_H_UGA_Results_2014.pdf)	I. District Health Services: **General Hospitals** provide preventive and general medical and surgical services, and limited specialist services. **Health Centres IV** have a provision for an operating theatre, in-patient and laboratory services, and is a referral facility for 20-30 level II and III HCs under its jurisdiction. **Health Centres III** offer basic lab services, maternity care, and inpatient care and are staffed by nurse aides, qualified nurses and clinical officers. **Health Centres II** are the lowest level of formal health care facilities and provide basic outpatient services	• 21 Clinics • 2217 Health Centres II • 1242 Health Centres III • 191 Health Centres IV • 110 Other Hospitals	• 831 Clinics • 2941 Health Centres II • 1239 Health Centres III • 197 Health Centres IV • 144 General Hospitals
	II. **Regional Referral Hospitals** are in each of the 14 health zones and provide referral services and supportive supervision to the district hospitals. Services provided include specialised medical and surgical care, basic research, and training of nurses and paramedical officers	• 9 Regional Referral Hospitals	• 14 Regional Referral Hospitals
	III. **National Referral Hospitals** are autonomous and provide referral services to the general and regional hospitals countrywide. They provide highly specialised medical and surgical services, advanced diagnostic services and advanced research and training for all cadres of medical professionals	• 2 National Referral Hospitals	• 2 National Referral Hospitals
Zambia (https://www.uhc2030.org/fileadmin/uploads/ihp/Documents/Country_Pages/Zambia/ZambiaNHSP2011to2015final..pdf) (http://apps.who.int/healthinfo/systems/datacatalog/index.php/catalog/36/reports)	I. Community: **Health Centres** form the backbone of primary health care in Zambia and serve a catchment population of 30,000-50,000 people in urban areas and 10,000 in rural areas. They are staffed by a clinical officer, nurse, or environmental health technician. **Health Posts** offer basic first aid rather than curative services	• 52 Clinics • 895 Health Posts • 107 Rural Health Centres • 118 Health Centres	• 953 Health Posts • 1839 Health Centres
	II. District: First Level Referral Hospitals (**District Hospitals)** provide diagnostic, medical, surgical and obstetric services, and all clinical services referred from health centres	• 68 Level 1 Hospitals	• 99 Level 1 Hospitals
	III. Provincial: Second Level Referral Hospitals (**Provincial** or **General Hospitals)** offer internal medicine, general surgery, pediatrics, obstetrics and gynecology, dental, psychiatry and intensive care services. They handle referrals from first level institutions, including technical back up and training services	• 19 Level 2 Hospitals	• 34 Level 2 Hospitals
	IV. National: Third Level Hospitals (**National/Specialist** or **Tertiary Hospitals**) are the highest referral hospitals and have sub-specializations in intensive care, surgery, obstetrics, pediatrics, internal medicine, gynecology, psychiatry, training and research	• 4 Level 3 Hospitals	• 8 Level 3 Hospitals
Zanzibar (http://www.tzdpg.or.tz/fileadmin/documents/dpg_internal/key_national_processes_%25_priority_areas_for_2009/MKUKUTA_MKUZA_Review_and_drafting/MKUZA_Studies/Health_study.pdf) (https://www.google.com/url?sa=t&rct=j&q=&esrc=s&source=web&cd=1&cad=rja&uact=8&ved=2ahUKEwjG2dmIk77iAhWMnxQKHUuoBIEQFjAAegQIBBAC&url=http%3A%2F%2Ftanzania.um.dk%2F~%2Fmedia%2FTanzania%2FDocuments%2FHealth%2FZanzibar%2520Strategic%2520Plan%252014%2520-%252018.pdf%3Fla%3Den&usg=AOvVaw3VMLPmQGTMK3Ftm02R_NqP)	I. **Dispensaries** and First and Second Line **Primary Health Care Units (PHCU** and **PHCU**+). **PHCUs** are the lowest level of health service provision, first point of care in the health system and provide basic outpatient services and outreach/community based health care services. **PHCUs + **offer facility-based delivery, basic laboratory services and dental services in addition to services offered by PHCUs	• 109 Primary Health Care Units • 28 Primary Health Care Units +	• 100 Primary Health Care Units • 34 Primary Health Care Units +
	II. **Primary Health Care Centres (Cottage hospitals)** provide basic inpatient care in areas far from other health facilities. They provide in-patient medical and basic surgical services, ambulatory services, psychiatric assessment and comprehensive emergency obstetric care including C-sections	• 4 Primary Health Care Centres	• 4 Primary Health Care Centres
	III. **District Hospitals** are all in Pemba and provide referral services to level I and II facilities	• 3 District Hospitals	• 3 District Hospitals
	IV. Special and Tertiary Hospitals: there is a **Referral Hospital** offers specialist tertiary services nationally and provides primary and secondary health care.	• 1 Tertiary Hospitals	• 1 Tertiary Hospitals
Zimbabwe (https://malariaelimination8.org/wp-content/uploads/2017/02/National%20Health%20Strategy%20for%20Zimbabwe%202016-2020.pdf) (https://www.hfgproject.org/wp-content/uploads/2015/02/Zimbabwe_Health_System_Assessment20101.pdf)	I. Primary: **Rural Health Centres** offer primary care services at the ward level and are the first point of contact with the health system in rural areas. They provide basic prevention, curative and maternity services	• 504 Rural Health Clinics • 563 Clinics	• 1243 Clinics • 307 Rural Health Centres • 15 Polyclinics
	II. Secondary/district: **District Hospitals** provide emergency, ambulatory and inpatient services in the districts and receives referrals from primary care facilities	• 108 Rural Hospitals • 47 District Hospitals	• 62 Rural Hospitals • 62 Mission Hospitals • 44 District Hospitals
	III. Tertiary/provincial: **Provincial Hospitals** in the rural provinces provide ambulatory and inpatient specialist services and receive referrals from district hospitals	• 1 Designated District/Provincial Hospital • 7 Provincial Hospitals	• 8 Provincial Hospitals
	IV. Quaternary/central: **Central Teaching Hospitals** are the highest referral facilities offering specialist medical services. Located in Harare, Bulawayo and Chitungwiza	• 6 Central Hospitals	• 6 Central Hospitals

Other health sector reports were used in place of HSSPs for Equatorial Guinea, Nigeria and Sao Tome & Principe.

^a^Includes district, regional and central hospitals.

^b^Includes private health centres.

^c^Includes public and private facilities for the 3 levels of delivery.

^d^Includes community, secondary and tertiary hospitals.

Abbreviations

NR -No Records.

CHW - Community Health Worker.

EPI - Expanded Program on ImmunizationAbbreviations.

NR -No Records.

CHW - Community Health Worker.
